# *rac*-Ethyl 4-hy­droxy-4-trifluoro­methyl-6-(2,4,5-trimeth­oxy­phen­yl)-2-thio-1,3-diazinane-5-carboxyl­ate

**DOI:** 10.1107/S1600536812041013

**Published:** 2012-10-10

**Authors:** Yong-Qiang Li, Zhi-Yu Ju

**Affiliations:** a State Key Laboratory of Elemento-Organic Chemistry, Institute of Elemento-Organic Chemistry, Nankai University, Tianjin, 300071, People’s Republic of China; b College of Chemistry and Chemical Engineering, Xuchang University, Xuchang, Henan Province, 461000, People’s Republic of China

## Abstract

In the title compound, C_17_H_21_F_3_N_2_O_6_S, the hexa­hydro­pyrimidine ring adopts a half-chair conformation: the mean plane formed by the ring atoms excluding the C atom bonded to the eth­oxy­carbonyl group has an r.m.s. deviation of 0.0427 Å and forms a dihedral angle of 66.41 (5)° with the benzene ring. The mol­ecular conformation is stabilized by an intra­molecular hydroxyl O—H⋯O_carbox­yl_ hydrogen bond, generating an *S*(6) ring. In the crystal, pairs of N—H⋯S and N–H⋯O hydrogen bonds give rise to the formation of two-dimensional networks lying parallel to the *ab* plane, which incorporate graph-set motifs *R* ^2^_2_(8) and *R* ^2^_2_(16), respectively.

## Related literature

For the bioactivity of dihydro­pyrimidines, see: Brier *et al.* (2004 ▶); Cochran *et al.* (2005 ▶); Moran *et al.* (2007 ▶); Zorkun *et al.* (2006 ▶) and for the bioactivity of organofluorine compounds, see: Hermann *et al.* (2003 ▶); Ulrich (2004 ▶). For the original Biginelli synthesis, see: Biginelli (1893 ▶). For a related structure, see: Li *et al.* (2011 ▶). For graph-set analysis, see: Bernstein *et al.* (1995 ▶).
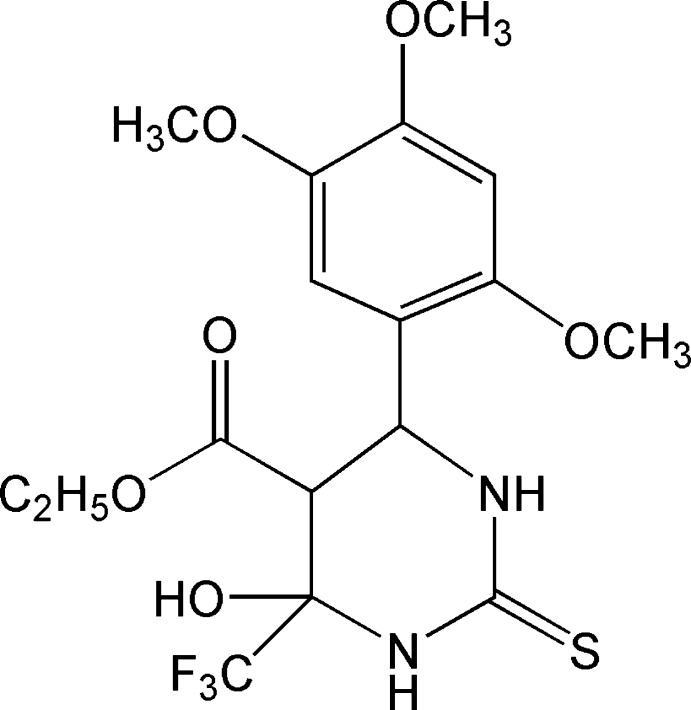


## Experimental

### 

#### Crystal data

C_17_H_21_F_3_N_2_O_6_S
*M* *_r_* = 438.42
Triclinic, 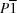

*a* = 9.5070 (8) Å
*b* = 9.9040 (8) Å
*c* = 11.4710 (13) Å
α = 71.582 (13)°
β = 76.740 (16)°
γ = 79.743 (15)°
*V* = 990.89 (19) Å^3^
*Z* = 2
Mo *K*α radiation
μ = 0.23 mm^−1^
*T* = 113 K
0.28 × 0.22 × 0.20 mm

#### Data collection

Rigaku Saturn724 CCD-detector diffractometer
Absorption correction: multi-scan (*REQAB*; Jacobson, 1998) ▶ *T*_min_ = 0.939, *T*_max_ = 0.956
13891 measured reflections
5290 independent reflections
3175 reflections with *I* > 2σ(*I*)
*R*_int_ = 0.049

#### Refinement

*R*[*F* ^2^ > 2σ(*F* ^2^)] = 0.033
*wR*(*F* ^2^) = 0.080
*S* = 0.90
5290 reflections
279 parameters
H atoms treated by a mixture of independent and constrained refinement
Δρ_max_ = 0.33 e Å^−3^
Δρ_min_ = −0.26 e Å^−3^

### 

Data collection: *CrystalClear* (Rigaku/MSC, 2009 ▶); cell refinement: *CrystalClear*; data reduction: *CrystalClear*; program(s) used to solve structure: *SHELXS97* (Sheldrick, 2008 ▶); program(s) used to refine structure: *SHELXL97* (Sheldrick, 2008 ▶); molecular graphics: *CrystalStructure* (Rigaku/MSC, 2009 ▶); software used to prepare material for publication: *CrystalStructure*.

## Supplementary Material

Crystal structure: contains datablock(s) shelxl, I. DOI: 10.1107/S1600536812041013/zs2236sup1.cif

Structure factors: contains datablock(s) I. DOI: 10.1107/S1600536812041013/zs2236Isup2.hkl

Supplementary material file. DOI: 10.1107/S1600536812041013/zs2236Isup3.cml

Additional supplementary materials:  crystallographic information; 3D view; checkCIF report

## Figures and Tables

**Table 1:** Hydrogen-bond geometry (Å, °)

*D*—H⋯*A*	*D*—H	H⋯*A*	*D*⋯*A*	*D*—H⋯*A*
O1—H1⋯O2	0.820 (15)	2.103 (16)	2.8055 (15)	143.5 (15)
N2—H2⋯O5^i^	0.824 (15)	2.129 (15)	2.9521 (15)	176.6 (15)
N1—H1*A*⋯S1^ii^	0.834 (15)	2.526 (16)	3.3427 (13)	166.3 (15)

Symmetry codes: (i) 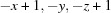; (ii) 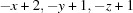.

**Fig. 1.**
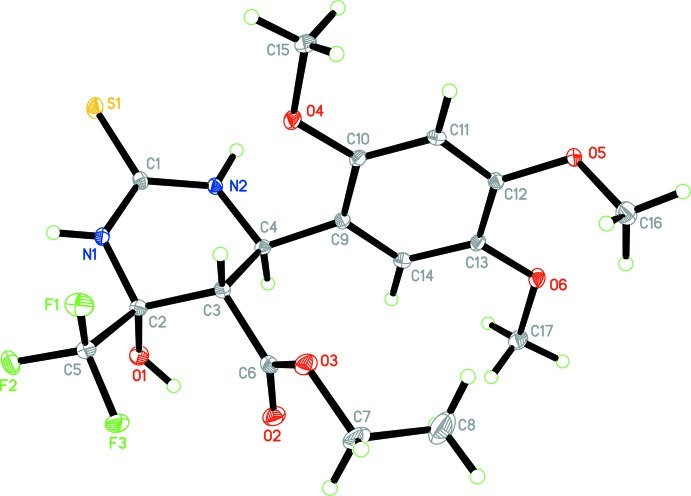
Molecular conformation and atom numbering scheme for the title compound, with displacement ellipsoids drawn at the 30% probability level.

**Fig. 2.**
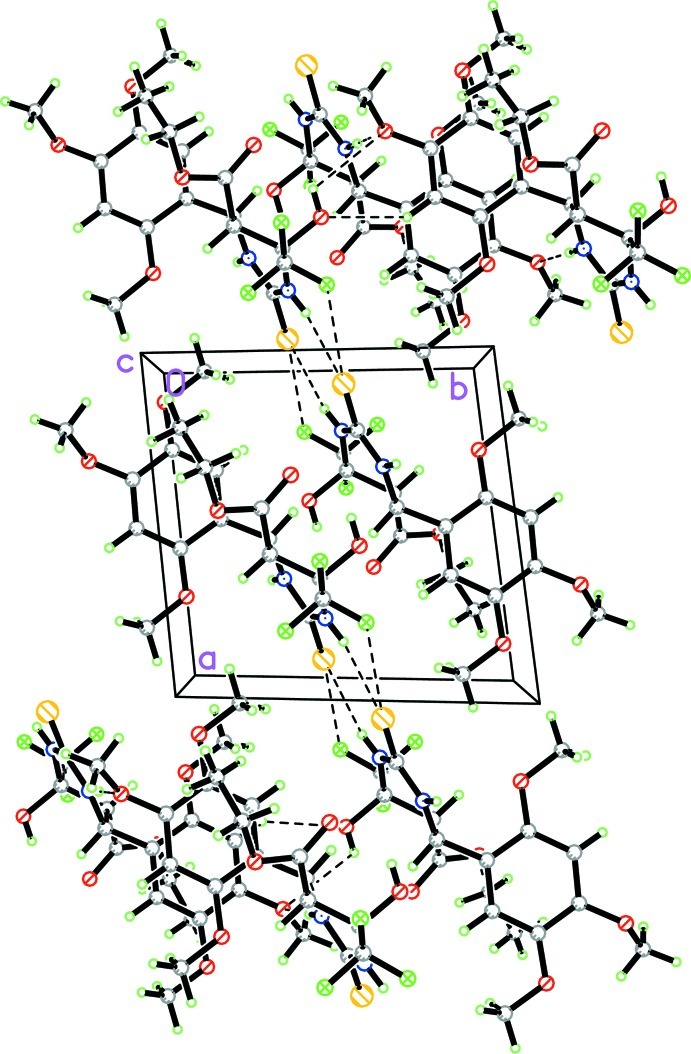
The packing of the title compound in the unit cell viewed down the c axis, with hydrogen bonds shown as dashed lines.

## supplementary crystallographic information

### Comment

The Biginelli reaction, the direct synthesis of dihydropyrimidinones by the one-pot condensation of aldehydes, urea or thiourea, was first reported more than a century ago (Biginelli, 1893). Dihydropyrimidine (DHPM) derivatives can be used as potential calcium channel blockers (Zorkun *et al.*, 2006), inhibitors of mitotic kinesin Eg5 for treating cancer (Cochran *et al.*, 2005; Brier *et al.*, 2004) and as TRPA1 modulators for treating pain (Moran *et al.*, 2007). In addition, compounds that contain fluorine have special bioactivity, e.g. flumioxazin is a widely used herbicide (Hermann *et al.*, 2003; Ulrich, 2004). This led us to focus our attention on the synthesis and bioactivity of these important fused perfluoroalkylated heterocyclic compounds. During the synthesis of DHPM derivatives, the title compound, an intermediate C_17_H_21_F_3_N_2_O_6_S was isolated and the structure confirmed by X-ray diffraction.

In the structure of the title molecule, the hexahydropyrimidine ring adopts a half-chair conformation, the mean plane formed by the ring atoms excluding the C atom bonded to the ethoxy carbonyl group has an r.m.s. deviation of 0.0427 Å, with a dihedral angle of 66.41 (5)° between the this plane and the benzene ring. The molecular conformation is stabilized by an intramolecular hydroxyl O—H···O_carboxyl_ hydrogen bond (Table 1), generating an *S*(6) ring. In the crystal structure, intermolecular cyclic N—H···S, and N—H···O hydrogen-bonding interactions [graph sets *R*^2^_2_(8) and *R*^2^_2_(16), respectively (Bernstein *et al.*, 1995)], together with a short hydroxyl O—H···O interaction give a two-dimensional structure (Fig. 2). For the crystal structure of a compound related to the title compound, see Li *et al.* (2011).

### Experimental

The title compound was synthesized by refluxing for 3 h a stirred solution of 2,4,5-trimethoxybenzaldehyde (0.98 g, 5 mmol), ethyl 4,4,4-trifluoro-3-oxobutanoate (1.11 g, 6 mmol) and thiourea (0.57 g, 7.5 mmol) in 5 ml of anhydrous ethanol, the reaction catalyzed by sulfamic acid (0.15 g). The solvent was evaporated *in vacuo* and the residue was washed with water. The title compound was recrystallized from 50% aqueous ethanol and single crystals were obtained by slow room-temperature evaporation of the solution.

### Refinement

Hydrogen atoms involved in hydrogen-bonding interactions were located by difference methods and their positional and isotropic displacement parameters were refined. Other H atoms were placed in calculated positions, with C—H(aromatic) = 0.95 Å and C—H(aliphatic) = 0.98 Å, 0.99 Å or 1.00 Å and treated as riding, with *U*_iso_(H) = 1.2*U*_eq_(C).

### Crystal data

**Table:**

C_17_H_21_F_3_N_2_O_6_S	*Z* = 2
*M**_r_* = 438.42	*F*(000) = 456
Triclinic, *P*1	*D*_x_ = 1.469 Mg m^−^^3^
Hall symbol: -P 1	Mo *K*α radiation, λ = 0.71075 Å
*a* = 9.5070 (8) Å	Cell parameters from 3446 reflections
*b* = 9.9040 (8) Å	θ = 1.9–29.2°
*c* = 11.4710 (13) Å	µ = 0.23 mm^−^^1^
α = 71.582 (13)°	*T* = 113 K
β = 76.740 (16)°	Prism, colorless
γ = 79.743 (15)°	0.28 × 0.22 × 0.20 mm
*V* = 990.89 (19) Å^3^	

### Data collection

**Table:**

Rigaku Saturn724 CCD-detector diffractometer	5290 independent reflections
Radiation source: rotating anode	3175 reflections with *I* > 2σ(*I*)
Multilayer monochromator	*R*_int_ = 0.049
Detector resolution: 14.222 pixels mm^-1^	θ_max_ = 29.1°, θ_min_ = 1.9°
ω scans	*h* = −12→13
Absorption correction: multi-scan (*REQAB*; Jacobson, 1998)	*k* = −13→13
*T*_min_ = 0.939, *T*_max_ = 0.956	*l* = −15→15
13891 measured reflections	

### Refinement

**Table:**

Refinement on *F*^2^	Secondary atom site location: difference Fourier map
Least-squares matrix: full	Hydrogen site location: inferred from neighbouring sites
*R*[*F*^2^ > 2σ(*F*^2^)] = 0.033	H atoms treated by a mixture of independent and constrained refinement
*wR*(*F*^2^) = 0.080	*w* = 1/[σ^2^(*F*_o_^2^) + (0.0266*P*)^2^ + ] where *P* = (*F*_o_^2^ + 2*F*_c_^2^)/3
*S* = 0.90	(Δ/σ)_max_ = 0.001
5290 reflections	Δρ_max_ = 0.33 e Å^−^^3^
279 parameters	Δρ_min_ = −0.26 e Å^−^^3^
0 restraints	Extinction correction: *SHELXL97*, Fc^*^=kFc[1+0.001xFc^2^λ^3^/sin(2θ)]^-1/4^
Primary atom site location: structure-invariant direct methods	Extinction coefficient: 0.0101 (11)

### Special details

**Table:**

Geometry. All e.s.d.'s (except the e.s.d. in the dihedral angle between two l.s. planes) are estimated using the full covariance matrix. The cell e.s.d.'s are taken into account individually in the estimation of e.s.d.'s in distances, angles and torsion angles; correlations between e.s.d.'s in cell parameters are only used when they are defined by crystal symmetry. An approximate (isotropic) treatment of cell e.s.d.'s is used for estimating e.s.d.'s involving l.s. planes.
Refinement. Refinement of *F*^2^ against ALL reflections. The weighted *R*-factor *wR* and goodness of fit *S* are based on *F*^2^, conventional *R*-factors *R* are based on *F*, with *F* set to zero for negative *F*^2^. The threshold expression of *F*^2^ > σ(*F*^2^) is used only for calculating *R*-factors(gt) *etc*. and is not relevant to the choice of reflections for refinement. *R*-factors based on *F*^2^ are statistically about twice as large as those based on *F*, and *R*- factors based on ALL data will be even larger.

### Fractional atomic coordinates and isotropic or equivalent isotropic displacement parameters (Å^2^)

**Table:**

	*x*	*y*	*z*	*U*_iso_*/*U*_eq_	
S1	0.92027 (4)	0.42289 (4)	0.38162 (3)	0.02116 (10)	
F1	0.81454 (9)	0.32810 (9)	0.86049 (8)	0.0272 (2)	
F2	0.79020 (9)	0.55959 (9)	0.79756 (8)	0.0284 (2)	
F3	0.61189 (9)	0.44824 (10)	0.91797 (8)	0.0276 (2)	
O1	0.56858 (11)	0.56726 (10)	0.67450 (10)	0.0200 (2)	
O2	0.35960 (10)	0.39204 (11)	0.83456 (10)	0.0237 (2)	
O3	0.46465 (10)	0.17323 (11)	0.92763 (9)	0.0221 (2)	
O4	0.72397 (10)	0.03795 (10)	0.63698 (9)	0.0195 (2)	
O5	0.31176 (10)	−0.21345 (10)	0.69741 (9)	0.0183 (2)	
O6	0.13106 (10)	0.03586 (10)	0.68092 (10)	0.0196 (2)	
N1	0.78623 (12)	0.44308 (13)	0.60651 (11)	0.0160 (3)	
N2	0.67147 (12)	0.33257 (13)	0.51166 (11)	0.0148 (3)	
C1	0.78416 (14)	0.39765 (14)	0.50737 (12)	0.0144 (3)	
C2	0.66487 (14)	0.44157 (14)	0.70770 (13)	0.0154 (3)	
C3	0.59354 (14)	0.30412 (14)	0.73587 (12)	0.0143 (3)	
H3	0.6653	0.2186	0.7617	0.017*	
C4	0.54789 (14)	0.30384 (14)	0.61551 (12)	0.0139 (3)	
H4	0.4682	0.3838	0.5970	0.017*	
C5	0.72143 (15)	0.44432 (16)	0.82155 (13)	0.0194 (3)	
C6	0.45866 (15)	0.29678 (15)	0.83747 (13)	0.0177 (3)	
C7	0.34010 (16)	0.15335 (18)	1.03094 (14)	0.0295 (4)	
H7A	0.3011	0.2466	1.0472	0.035*	
H7B	0.3722	0.0874	1.1075	0.035*	
C8	0.22227 (18)	0.0932 (2)	1.00195 (17)	0.0471 (5)	
H8A	0.1833	0.1628	0.9315	0.057*	
H8B	0.1441	0.0732	1.0754	0.057*	
H8C	0.2624	0.0043	0.9801	0.057*	
C9	0.48928 (14)	0.16512 (14)	0.63245 (12)	0.0139 (3)	
C10	0.57798 (14)	0.03450 (15)	0.64410 (12)	0.0148 (3)	
C11	0.51657 (14)	−0.09063 (14)	0.66579 (12)	0.0156 (3)	
H11	0.5770	−0.1790	0.6717	0.019*	
C12	0.36760 (14)	−0.08670 (14)	0.67880 (13)	0.0154 (3)	
C13	0.27734 (14)	0.04244 (15)	0.66821 (12)	0.0150 (3)	
C14	0.33970 (14)	0.16737 (15)	0.64445 (12)	0.0152 (3)	
H14	0.2792	0.2560	0.6362	0.018*	
C15	0.81956 (14)	−0.09137 (15)	0.63483 (14)	0.0202 (3)	
H15A	0.7976	−0.1643	0.7152	0.024*	
H15B	0.9207	−0.0719	0.6201	0.024*	
H15C	0.8057	−0.1261	0.5677	0.024*	
C16	0.21996 (16)	−0.26397 (16)	0.81787 (13)	0.0259 (4)	
H16A	0.2755	−0.2799	0.8841	0.031*	
H16B	0.1859	−0.3540	0.8236	0.031*	
H16C	0.1361	−0.1922	0.8276	0.031*	
C17	0.03752 (14)	0.16588 (16)	0.68146 (15)	0.0240 (4)	
H17A	0.0532	0.2022	0.7471	0.029*	
H17B	−0.0641	0.1473	0.6976	0.029*	
H17C	0.0594	0.2372	0.6001	0.029*	
H1	0.4866 (17)	0.5528 (17)	0.7147 (15)	0.037 (6)*	
H1A	0.8556 (17)	0.4884 (18)	0.5985 (16)	0.040 (5)*	
H2	0.6770 (15)	0.2958 (16)	0.4553 (14)	0.025 (5)*	

### Atomic displacement parameters (Å^2^)

**Table:**

	*U*^11^	*U*^22^	*U*^33^	*U*^12^	*U*^13^	*U*^23^
S1	0.0211 (2)	0.0299 (2)	0.01504 (19)	−0.01212 (17)	0.00076 (15)	−0.00803 (16)
F1	0.0276 (5)	0.0317 (5)	0.0234 (5)	0.0066 (4)	−0.0116 (4)	−0.0100 (4)
F2	0.0321 (5)	0.0333 (5)	0.0273 (5)	−0.0162 (4)	−0.0006 (4)	−0.0159 (4)
F3	0.0227 (5)	0.0429 (6)	0.0209 (5)	−0.0075 (4)	0.0038 (4)	−0.0179 (4)
O1	0.0177 (6)	0.0143 (5)	0.0266 (6)	0.0009 (4)	−0.0044 (5)	−0.0052 (4)
O2	0.0185 (6)	0.0235 (6)	0.0266 (6)	0.0010 (5)	−0.0002 (5)	−0.0084 (5)
O3	0.0244 (6)	0.0220 (6)	0.0160 (5)	−0.0049 (5)	0.0024 (4)	−0.0030 (4)
O4	0.0115 (5)	0.0170 (5)	0.0301 (6)	−0.0007 (4)	−0.0038 (4)	−0.0077 (5)
O5	0.0183 (5)	0.0175 (5)	0.0205 (6)	−0.0078 (4)	0.0026 (4)	−0.0088 (4)
O6	0.0119 (5)	0.0169 (5)	0.0300 (6)	−0.0012 (4)	−0.0047 (4)	−0.0062 (5)
N1	0.0140 (6)	0.0199 (7)	0.0161 (6)	−0.0065 (5)	−0.0006 (5)	−0.0072 (5)
N2	0.0160 (6)	0.0169 (6)	0.0127 (6)	−0.0054 (5)	−0.0009 (5)	−0.0053 (5)
C1	0.0166 (7)	0.0116 (7)	0.0157 (7)	−0.0017 (6)	−0.0060 (6)	−0.0027 (6)
C2	0.0160 (7)	0.0155 (7)	0.0151 (7)	−0.0025 (6)	−0.0016 (6)	−0.0053 (6)
C3	0.0134 (7)	0.0143 (7)	0.0157 (7)	−0.0011 (6)	−0.0026 (6)	−0.0052 (6)
C4	0.0123 (7)	0.0135 (7)	0.0155 (7)	−0.0001 (6)	−0.0022 (6)	−0.0043 (6)
C5	0.0174 (7)	0.0210 (8)	0.0201 (8)	−0.0030 (6)	0.0004 (6)	−0.0086 (6)
C6	0.0197 (8)	0.0185 (8)	0.0173 (7)	−0.0047 (6)	−0.0030 (6)	−0.0077 (6)
C7	0.0302 (9)	0.0356 (10)	0.0165 (8)	−0.0071 (8)	0.0065 (7)	−0.0050 (7)
C8	0.0394 (11)	0.0665 (14)	0.0341 (11)	−0.0269 (10)	0.0071 (9)	−0.0112 (10)
C9	0.0158 (7)	0.0144 (7)	0.0114 (7)	−0.0033 (6)	−0.0014 (5)	−0.0034 (6)
C10	0.0117 (7)	0.0191 (8)	0.0140 (7)	−0.0027 (6)	−0.0012 (6)	−0.0055 (6)
C11	0.0159 (7)	0.0142 (7)	0.0165 (7)	0.0016 (6)	−0.0026 (6)	−0.0062 (6)
C12	0.0173 (7)	0.0162 (7)	0.0142 (7)	−0.0059 (6)	−0.0006 (6)	−0.0059 (6)
C13	0.0116 (7)	0.0187 (7)	0.0151 (7)	−0.0032 (6)	−0.0014 (5)	−0.0055 (6)
C14	0.0158 (7)	0.0149 (7)	0.0149 (7)	0.0000 (6)	−0.0031 (6)	−0.0049 (6)
C15	0.0148 (7)	0.0203 (8)	0.0245 (8)	0.0015 (6)	−0.0027 (6)	−0.0078 (6)
C16	0.0362 (9)	0.0217 (8)	0.0180 (8)	−0.0105 (7)	0.0011 (7)	−0.0035 (6)
C17	0.0134 (7)	0.0235 (9)	0.0345 (9)	0.0012 (6)	−0.0035 (7)	−0.0102 (7)

### Geometric parameters (Å, º)

**Table:**

S1—C1	1.6878 (14)	C4—C9	1.5133 (18)
F1—C5	1.3422 (16)	C4—H4	1.0000
F2—C5	1.3384 (16)	C7—C8	1.501 (2)
F3—C5	1.3392 (15)	C7—H7A	0.9900
O1—C2	1.4096 (16)	C7—H7B	0.9900
O1—H1	0.820 (15)	C8—H8A	0.9800
O2—C6	1.2068 (16)	C8—H8B	0.9800
O3—C6	1.3314 (17)	C8—H8C	0.9800
O3—C7	1.4619 (16)	C9—C14	1.3937 (17)
O4—C10	1.3775 (15)	C9—C10	1.3988 (19)
O4—C15	1.4360 (15)	C10—C11	1.3910 (18)
O5—C12	1.3854 (15)	C11—C12	1.3844 (17)
O5—C16	1.4445 (16)	C11—H11	0.9500
O6—C13	1.3758 (15)	C12—C13	1.3949 (19)
O6—C17	1.4296 (16)	C13—C14	1.3895 (19)
N1—C1	1.3538 (17)	C14—H14	0.9500
N1—C2	1.4352 (17)	C15—H15A	0.9800
N1—H1A	0.834 (15)	C15—H15B	0.9800
N2—C1	1.3295 (16)	C15—H15C	0.9800
N2—C4	1.4640 (16)	C16—H16A	0.9800
N2—H2	0.824 (15)	C16—H16B	0.9800
C2—C5	1.5315 (19)	C16—H16C	0.9800
C2—C3	1.5388 (18)	C17—H17A	0.9800
C3—C6	1.5172 (18)	C17—H17B	0.9800
C3—C4	1.5403 (18)	C17—H17C	0.9800
C3—H3	1.0000		
			
C2—O1—H1	109.2 (12)	C8—C7—H7B	109.4
C6—O3—C7	116.46 (11)	H7A—C7—H7B	108.0
C10—O4—C15	117.60 (10)	C7—C8—H8A	109.5
C12—O5—C16	114.69 (11)	C7—C8—H8B	109.5
C13—O6—C17	116.33 (10)	H8A—C8—H8B	109.5
C1—N1—C2	123.14 (12)	C7—C8—H8C	109.5
C1—N1—H1A	115.4 (12)	H8A—C8—H8C	109.5
C2—N1—H1A	120.3 (12)	H8B—C8—H8C	109.5
C1—N2—C4	125.74 (12)	C14—C9—C10	119.20 (12)
C1—N2—H2	116.9 (10)	C14—C9—C4	118.14 (12)
C4—N2—H2	116.8 (10)	C10—C9—C4	122.54 (12)
N2—C1—N1	118.08 (12)	O4—C10—C11	123.52 (12)
N2—C1—S1	120.77 (11)	O4—C10—C9	116.59 (12)
N1—C1—S1	121.16 (10)	C11—C10—C9	119.86 (12)
O1—C2—N1	108.97 (11)	C12—C11—C10	120.18 (13)
O1—C2—C5	107.58 (11)	C12—C11—H11	119.9
N1—C2—C5	108.28 (11)	C10—C11—H11	119.9
O1—C2—C3	112.87 (11)	C11—C12—O5	118.11 (12)
N1—C2—C3	108.27 (11)	C11—C12—C13	120.75 (12)
C5—C2—C3	110.77 (12)	O5—C12—C13	121.09 (12)
C6—C3—C2	112.14 (11)	O6—C13—C14	124.72 (12)
C6—C3—C4	108.08 (11)	O6—C13—C12	116.52 (12)
C2—C3—C4	108.03 (11)	C14—C13—C12	118.76 (12)
C6—C3—H3	109.5	C13—C14—C9	121.24 (13)
C2—C3—H3	109.5	C13—C14—H14	119.4
C4—C3—H3	109.5	C9—C14—H14	119.4
N2—C4—C9	112.66 (11)	O4—C15—H15A	109.5
N2—C4—C3	109.02 (10)	O4—C15—H15B	109.5
C9—C4—C3	111.13 (11)	H15A—C15—H15B	109.5
N2—C4—H4	108.0	O4—C15—H15C	109.5
C9—C4—H4	108.0	H15A—C15—H15C	109.5
C3—C4—H4	108.0	H15B—C15—H15C	109.5
F2—C5—F3	106.90 (11)	O5—C16—H16A	109.5
F2—C5—F1	107.50 (11)	O5—C16—H16B	109.5
F3—C5—F1	107.21 (11)	H16A—C16—H16B	109.5
F2—C5—C2	111.95 (12)	O5—C16—H16C	109.5
F3—C5—C2	110.97 (12)	H16A—C16—H16C	109.5
F1—C5—C2	112.04 (11)	H16B—C16—H16C	109.5
O2—C6—O3	125.39 (13)	O6—C17—H17A	109.5
O2—C6—C3	123.22 (13)	O6—C17—H17B	109.5
O3—C6—C3	111.39 (12)	H17A—C17—H17B	109.5
O3—C7—C8	111.32 (13)	O6—C17—H17C	109.5
O3—C7—H7A	109.4	H17A—C17—H17C	109.5
C8—C7—H7A	109.4	H17B—C17—H17C	109.5
O3—C7—H7B	109.4		
			
C4—N2—C1—N1	−0.5 (2)	C4—C3—C6—O2	−66.66 (17)
C4—N2—C1—S1	179.59 (10)	C2—C3—C6—O3	−127.98 (12)
C2—N1—C1—N2	−8.5 (2)	C4—C3—C6—O3	113.05 (13)
C2—N1—C1—S1	171.37 (10)	C6—O3—C7—C8	86.53 (17)
C1—N1—C2—O1	−84.44 (15)	N2—C4—C9—C14	−131.29 (13)
C1—N1—C2—C5	158.83 (13)	C3—C4—C9—C14	106.04 (14)
C1—N1—C2—C3	38.67 (17)	N2—C4—C9—C10	52.74 (18)
O1—C2—C3—C6	−56.23 (15)	C3—C4—C9—C10	−69.94 (16)
N1—C2—C3—C6	−176.95 (11)	C15—O4—C10—C11	8.80 (19)
C5—C2—C3—C6	64.47 (15)	C15—O4—C10—C9	−173.30 (12)
O1—C2—C3—C4	62.77 (14)	C14—C9—C10—O4	−177.16 (12)
N1—C2—C3—C4	−57.94 (14)	C4—C9—C10—O4	−1.23 (19)
C5—C2—C3—C4	−176.53 (11)	C14—C9—C10—C11	0.8 (2)
C1—N2—C4—C9	−146.08 (13)	C4—C9—C10—C11	176.76 (12)
C1—N2—C4—C3	−22.23 (18)	O4—C10—C11—C12	176.31 (12)
C6—C3—C4—N2	171.55 (11)	C9—C10—C11—C12	−1.5 (2)
C2—C3—C4—N2	50.00 (14)	C10—C11—C12—O5	178.53 (12)
C6—C3—C4—C9	−63.69 (14)	C10—C11—C12—C13	1.2 (2)
C2—C3—C4—C9	174.76 (10)	C16—O5—C12—C11	114.47 (14)
O1—C2—C5—F2	−59.19 (14)	C16—O5—C12—C13	−68.16 (17)
N1—C2—C5—F2	58.44 (15)	C17—O6—C13—C14	−6.35 (19)
C3—C2—C5—F2	177.02 (11)	C17—O6—C13—C12	174.47 (12)
O1—C2—C5—F3	60.15 (14)	C11—C12—C13—O6	179.16 (12)
N1—C2—C5—F3	177.78 (11)	O5—C12—C13—O6	1.86 (19)
C3—C2—C5—F3	−63.64 (15)	C11—C12—C13—C14	−0.1 (2)
O1—C2—C5—F1	179.96 (10)	O5—C12—C13—C14	−177.37 (12)
N1—C2—C5—F1	−62.42 (14)	O6—C13—C14—C9	−179.80 (13)
C3—C2—C5—F1	56.17 (15)	C12—C13—C14—C9	−0.6 (2)
C7—O3—C6—O2	−0.1 (2)	C10—C9—C14—C13	0.3 (2)
C7—O3—C6—C3	−179.80 (11)	C4—C9—C14—C13	−175.86 (12)
C2—C3—C6—O2	52.32 (18)		

### Hydrogen-bond geometry (Å, º)

**Table:**

*D*—H···*A*	*D*—H	H···*A*	*D*···*A*	*D*—H···*A*
O1—H1···O2	0.820 (15)	2.103 (16)	2.8055 (15)	143.5 (15)
O1—H1···O5^i^	0.820 (15)	2.579 (16)	2.9876 (15)	112.2 (13)
N2—H2···O5^ii^	0.824 (15)	2.129 (15)	2.9521 (15)	176.6 (15)
N1—H1*A*···S1^iii^	0.834 (15)	2.526 (16)	3.3427 (13)	166.3 (15)

Symmetry codes: (i) *x*, *y*+1, *z*; (ii) −*x*+1, −*y*, −*z*+1; (iii) −*x*+2, −*y*+1, −*z*+1.
